# Provision of mental health care within primary care in Peru: A qualitative study exploring the perspectives of psychologists, primary health care providers, and patients

**DOI:** 10.12688/wellcomeopenres.13746.2

**Published:** 2018-04-30

**Authors:** Victoria Cavero, Francisco Diez-Canseco, Mauricio Toyama, Gustavo Flórez Salcedo, Alessandra Ipince, Ricardo Araya, J. Jaime Miranda

**Affiliations:** 1 CRONICAS Center of Excellence in Chronic Diseases, Universidad Peruana Cayetano Heredia, Lima, Peru; 2 Institute of Psychiatry, Psychology & Neuroscience, King’s College London, London, UK; 3 School of Medicine, Universidad Peruana Cayetano Heredia, Lima, Peru

**Keywords:** mental health, primary care, health services research, qualitative study

## Abstract

**Background:** This study aimed to understand the offer of mental health care at the primary care level, collecting the views of psychologists, primary health care providers (PHCPs), and patients, with a focus on health services in which patients attend regularly and who present a higher prevalence of mental disorders.

**Methods:** A qualitative study was conducted in antenatal care, tuberculosis, HIV/AIDS, and chronic diseases services from six primary health care centers. Semi-structured interviews were conducted with psychologists, PHCPs, and patients working in or attending the selected facilities.

**Results:** A total of 4 psychologists, 22 PHCPs, and 37 patients were interviewed. A high perceived need for mental health care was noted. PHCPs acknowledged the emotional impact physical health conditions have on their patients and mentioned that referral to psychologists was reserved only for serious problems. Their approach to emotional problems was providing emotional support (includes listening, talk about their patients’ feelings, provide advice). PHCPs identified system-level barriers about the specialized mental health care, including a shortage of psychologists and an overwhelming demand, which results in brief consultations and lack in continuity of care. Psychologists focus their work on individual consultations; however, consultations were brief, did not follow a standardized model of care, and most patients attend only once. Psychologists also mentioned the lack of collaborative work among other healthcare providers. Despite these limitations, interviewed patients declared that they were willing to seek specialized care if advised and considered the psychologist's care provided as helpful; however, they recognized the stigmatization related to seeking mental health care.

**Conclusions:** There is a perceived need of mental health care for primary care patients. To attend these needs, PHCPs provide emotional support and refer to psychology the most severe cases, while psychologists provide one-to-one consultations. Significant limitations in the care provided are discussed.

## Introduction

Worldwide, mental disorders are highly prevalent ^1^, and represent 10% of the global burden of disease ^2^. Likewise, in Peru, mental disorders have a lifetime prevalence of 26% ^3^ and are the leading cause of burden of disease in the country, accounting for 16% of all DALYs ^4^. However, mental disorders remain underdiagnosed and undertreated in Peru ^5^, where only 31% of people who expressed having a mental health problem in the past 12 months, sought mental health care ^3^. This situation is mainly explained by health systems’ barriers, as the insufficiency and inequitable distribution of resources, the small budget allocated to mental health up until recent years, and the poor integration of mental health care into primary care ^6^. However, this treatment gap might be also explained by the fact that many patients who report having a mental health problem do not seek specialized care. Reasons for not seeking mental health care include thinking that they can solve their problems by their own, not having enough time, considering their problem as not serious or important, not having money to pay a consultation, not knowing where to seek mental health care, or having bad previous experiences ^3^.

To address these limitations, on 2008 the WHO developed the Mental Health Gap Action Programme (mhGAP), which aimed to strengthen the commitment of governments and key institutions to increase the assignation of human and financial resources for mental health care in low- and middle-income countries ^7^. On 2010, the WHO developed the mhGAP Intervention Guide for the Management of Common Mental Disorders in Primary Care. This guide has been implemented in several countries, including Peru. Building upon these initiatives, there is an ongoing Peruvian Mental Health Reform that aims to integrate the mental health care at the primary care level services ^8, 9^. This reform proposes, among other things, that primary health care providers (PHCPs) detect and treat mental disorders in their patients (detection is through the use of a common mental disorders’ screening, the Self Report Questionnaire SRQ) ^9, 10^. This would reduce the mental health treatment gap, as evidence in other countries reports that many people requiring mental health care attend other primary care services ^11^. Nonetheless, such integration would carry some difficulties; for instance, some studies have found that PHCPs may not correctly identify mental disorders in their patients ^12^, or are reluctant to treat mental health problems because of feelings of incompetence, lack of time, or scarcity of human resources ^13^.

In this scenario, it would be important to better understand how mental health care is provided in primary care in order to ease the implementation of this health reform. We wondered how mental health care is provided in the Peruvian primary health care public system: How do PHCPs perceive their patients’ mental health? What do PHCPs and psychologists do to attend their patients’ mental health? What do patients say about the care received? What are the barriers and facilitators to provide mental health care in primary care?

To answer these questions, we conducted a qualitative study with the aim of understanding the offer of mental health care in primary care, collecting the perspectives of psychologists, PHCPs and patients who worked in and attended primary health services.

## Methods

### Study design

This study was conducted as part of a study called Integration of mental health into primary health care services, the Allillanchu Project ^14^. The aim of this project was to improve early detection, opportune referral, and access to treatment of patients with mental disorders attending primary health care services through the introduction of a screening for mental disorders into PHCPs quotidian routines and a SMS package to remind and motivate patients to seek mental health care. In this scenario, and prior to its implementation, a qualitative formative study was conducted to understand the offer of mental health services for patients with physical health conditions in primary health care services. This paper presents the results of this qualitative formative study.

### Study setting

The study was conducted in six public primary health care facilities in Lima, Peru’s capital, five operating under the Ministry of Health (MoH) and one as part of the Peruvian Social Security System (EsSalud), all of them serving low-income populations. Whereas the MoH serves all the Peruvian population, especially those of scarce resources, EsSalud tend to patients who currently have a formal job. These health centers were selected for the implementation of the Allillanchu Project by convenience. Some of the health services selected were not offered in all primary health care centers, as the chronic diseases and HIV/AIDS health services, thus the research team approached health care centers in which they were offered (chronic diseases services were only offered in the health centers from EsSalud, and the HIV/AIDS services, in very few primary health care centers of the MoH). Additionally, there were previous contact with some PHCPs, psychologists and stakeholders in such centers, which ease the implementation of the project. Even though we worked with only six health care centers, the study incorporated different health services, in different settings and from the two most widely used health systems (MoH and EsSalud), in order to collect the variety of primary care characteristics to better understand the mental health care provision in primary care. Within these facilities, a total of 17 ambulatory healthcare services were involved in the study: 5 antenatal care, 5 tuberculosis, 1 chronic diseases, 2 HIV-AIDS, and 4 psychology (see Table 1). These health services were selected because they tend to patients who show a high prevalence of depression and whose access to the health system is typically through primary care: pregnant women ^15– 17^, people living in poverty ^3^, and people with chronic conditions ^18, 19^, such as tuberculosis ^20^, HIV/AIDS ^21^, diabetes ^22^ and hypertension ^23^.

**Table 1.** Distribution of health services per health centers and health systems. Seventeen health services were involved in the study: Antenatal care, tuberculosis, HIV/AIDS, and chronic diseases services among six health care centers, five from the Ministry of Health and one from EsSalud.

	Ministry of Health (MoH)					EsSalud	Total
	Health center #1	Health center #2	Health center #3	Health center #4	Health center #5	Health center #6	
**Antenatal care**	1	1	1	1	0	1	**5**
**Tuberculosis**	1	1	1	1	0	1	**5**
**HIV/AIDS**	0	0	1	0	1	0	**2**
**Chronic diseases**	0	0	0	0	0	1	**1**
**Psychology**	1	1	0	1	0	1	**4**
**Total**	**3**	**3**	**3**	**3**	**1**	**4**	**17**

### Participants

Three groups of participants were included in the study: PHCPs, psychologists and patients of primary health care centers from the MoH and EsSalud. Even though psychologists belong to the category of PHCPs, they are presented separately in order to emphasize the difference in the mental health care provided by those specialized in mental health (psychologists) and those who are not (nurses, midwives). As most primary health care centers, those in which the study was conducted had only psychologists as specialized mental health professionals, thus psychiatrists were not included in the data collection process. PHCPs and psychologists were approached in their consulting rooms, and patients were invited to participate in the waiting rooms, before they enter to their consultations.

***Psychologists***. We invited 6 psychologists, one from each health center, to be interviewed. The selection criteria included being in the capacity to provide informed consent, being willing to participate, and to be working within a primary health care facility.

***Primary health care providers***. Within the six selected health centers, we proposed to interview 24 PHCPs (midwives, nurses and nurses technicians), 2 per health service: 10 from antenatal care, 10 from tuberculosis, 2 from HIV/AIDS, and 2 from chronic diseases services. When possible, we tried to interview personnel with opposite views toward the implementation of the Allillanchu project; that is, those who were willing to participate and those who deemed it as not relevant to their work. To participate, potential interviewees were to be in capacity of providing informed consent and to have been employed in these services in any of the selected health centers for at least three months.

***Primary health care patients***. We proposed to interview 36 primary health care patients of antenatal care (12), tuberculosis care (12), HIV/AIDS care (6), and chronic diseases care (6). This distribution reflects the higher number of antenatal care and tuberculosis services (operating in all health centers) compared to chronic diseases and HIV/AIDS services (operating only in one and two health centers, respectively). The inclusion criteria for ambulatory patients was to be aged 18 years or older and in capacity to provide informed consent, with a focus on those attending regularly (appointments or treatment continuation) to any of the selected primary care services, thus excluding new patients. Therefore, additional eligibility criteria were considered for specific health services. Pregnant women must have completed at least 3 antenatal care visits, and only patients with pulmonary tuberculosis were included and must have completed the second month of their treatment. Patients must also have a negative result on their sputum smear microscopy test, and be willing to be interviewed in an open area to avoid infecting the interviewers; HIV/AIDS and chronic diseases (with diabetes or hypertension) patients must have had their diagnosis for no less than 6 months prior to the interview.

### Data collection tools

Semi-structured interviews were conducted using interview guides and datasheets. Datasheets were used to collect sociodemographic information from the interviewees. Interview guides were designed for each group of participants, all with different sections. The patient’s interview guides had three main sections: 1) experience with the physical health condition and use of mental health services, 2) care received to treat their health condition, and 3) opinion regarding the Allillanchu project. For PHCPs we included: 1) functioning of the primary care services, 2) provision of mental health care in their health service and in the health center, and 3) opinion and suggestions regarding the Allillanchu project. Finally, for psychologists, the sections included were: 1) description of patients attending the mental health service, 2) access and referral of patients to mental health services, and 3) opinion and suggestions regarding the Allillanchu project.

### Data collection procedures

Data collection was conducted from September to December 2014. Patients were approached at the health care centers and invited to participate before or after their appointments. Those who consented were interviewed after their consultations. PHCPs and psychologists were personally invited to participate and an interview date was scheduled.

Interviews were conducted individually and face to face by trained research team members in collection of qualitative data, and were audio-recorded for further analysis, as consented.

### Data analysis

Audio-recorded interviews were transcribed verbatim, and then analyzed using the qualitative software ATLAS.ti 7.1 (Scientific Software Development GmbH, Berlin, Germany). Three members of the research team, who were involved in the interview and/or transcription process, worked together in the elaboration of the codebook. The intention was to develop one codebook that includes the voices of the different groups of participants, based on the topics explored in the interview guides and the information collected through the interviews. The research team members reached consensus on the definition of each category of the codebook, making clear what information should and should not be included in each. Thus, information belonging to one category would not be repeated in another. Then, the research team members read one transcript for each group of participants and run the codebook to prove if the categories were clear and responsive to the data. Since the codebook proved to be appropriate, the same team members proceeded to code the rest of the interviews. For the complete codebook, see Supplementary File 1. At the end of the coding process, the output of the codebook with its respective quotations were exported to verify common themes, select illustrative quotes, and select the most relevant information to write a final report.

### Ethical statement

The study protocol, data collection tools and informed consent forms were approved by the Institutional Review Board of the Universidad Peruana Cayetano Heredia (IRB), with project identification number 62021 and approval document 124-10-14, on April 21 ^st^, 2014. The directors of the primary health care centers also gave their assent for the study to be conducted. All participants provided their oral consent to be interviewed and audio-recorded. Since patients with tuberculosis and HIV/AIDS are frequently stigmatized, and PHCPs and psychologists were supposed to talk about their health services and the care they provide, the signature and name in a document may have felt like a source of concern for them, and could interfere with their willingness to participate. Thus, written informed consent was not obtained to assure that participants felt their privacy and confidentiality were respected. However, all participants received written information about their participation in the study and kept a copy of the informed consent form after consenting their participation. As the study did not involve an intervention, their signature was not mandatory. The IRB approved the oral consent as an adequate procedure.

## Results

Following the description of the study participants, results are organized in three main sections. First, an overview of the perceptions of PHCPs regarding their patients’ mental health is provided. This is followed by a description of the facilities’ provision of mental health care to primary care patients, offered by PHCPs and psychologists.

### Participants and facilities characteristics

Trained research team members conducted a total of 63 semi-instructed interviews (see Table 2). A total of 37 patients attending the antenatal care, tuberculosis, chronic diseases and HIV/AIDS ambulatory services participated in the study, for which the mean age was 35.8 (± 15.3 years), and 64.9% patients were female. The 22 interviewed PHCPs were midwives (12), nurses (5), and nurse technicians (5), all women, with a mean age of 45.6 (± 10.4 years).

All six facilities had a mental health service, with one or two psychologists working in each. Additionally, the EsSalud health center had a complementary medicine service, where patients from the chronic diseases service are referred to for additional care, and to practice relaxation exercises and interact with other people with similar conditions.

**Table 2.** Distribution of participants per health service, health center and health system. Sixty three participants were involved in the study. Thirty seven were patients, twenty two primary health care providers (PHCPs) and four psychologists. The table shows their distribution among health services, health centers and health systems.

Participants	Health care service	Ministry of Health (MoH)					EsSalud	Total	
		Health center #1	Health center #2	Health center #3	Health center #4	Health center #5	Health center #6		
**Patients**	**Antenatal care**	2	3	2	2	0	4	**13**	**37**
	**Tuberculosis**	2	2	2	2	0	4	**12**	
	**HIV/AIDS**	0	0	1	0	5	0	**6**	
	**Chronic diseases**	0	0	0	0	0	6	**6**	
**PHCPs**	**Antenatal care**	3	2	0	2	0	3	**10**	**22**
	**Tuberculosis**	2	2	2	0	0	2	**8**	
	**HIV/AIDS**	0	0	2	0	0	0	**2**	
	**Chronic diseases**	0	0	0	0	0	2	**2**	
**Psychologists**	**Psychology**	1	1	0	1	0	1	**4**	**4**
**Total**		**10**	**10**	**9**	**7**	**5**	**22**	**63**	

### What PHCPs think about their patients’ mental health

PHCPs interviewed perceived that patients’ physical health condition (i.e. pregnancy, diabetes, etc.) affect their mental health, mainly based on the following: patients are worried about having a physical health condition; they are afraid of the impact the pregnancy or the disease has on their lives, like changing their lifestyle; or when PHCPs see them sad or effectively cry during the consultations.

According to PHCPs, the emotional wellbeing of patients differ among pregnant women and patients living with a physical disease and may vary across time. For instance, midwives considered that pregnant women’s sadness, anxiety or worries are not a direct consequence of their pregnancy but rather an worsening of previous adverse situations that are intensified with their pregnancy experience, e.g. women who had a bad relationship with their couples and will bear the pregnancy on their own, or women that live in poverty and are concerned about how they will afford having a child.

*“There are many women that bear their pregnancy by themselves, because they are not always with their couples, so they are the most depressed patients, they can be anxious. How many of these women have been abandoned by their couples, they have left them and they (pregnant women) come with a huge burden, very sensitive.”* (Midwife, antenatal care service, Health center #6).

On the other hand, for patients with diabetes, hypertension, tuberculosis or HIV/AIDS, PHCPs considered that their mental health was mainly affected by the onset and experience of their physical health condition, regardless of previous events. This emotional impact is considered to be brought upon fear or concern for the negative consequences of living with their new physical condition, as well as the lifestyle changes or further health complications; or due to the fear of stigmatization for patients with tuberculosis or HIV/AIDS. However, PHCPs also believe patients improve their emotional wellbeing over time, once they had accepted their physical health condition and return to their regular activities. Finally, and similarly to pregnant women, PHCPs pointed out that some patients could be emotionally affected by problems they had before their diagnosis, and considered these problems to be intensified by the diagnosis of a physical health condition.

### Care provided by PHCPs to enhance their patients’ mental health

Despite PHCPs’ roles in the above mentioned services are focused on the physical care of their patients, actions addressing their patients’ mental health were also noted. Interviewees stated that one approach to attend their patients’ mental health was by providing information about their physical health condition, its consequences and treatment; motivating them to adhere to the treatment; solving doubts; and talking to the family, since it would prevent their patients from feeling overburdened thus attending their mental health.

“ *I try to help my patient, not only in the physical part of their disease, but also in the spiritual part, in their mental health, to feel good. (…) We help our patients mainly through education, raising awareness about the causes of their conditions, how they can manage their fears, and how to take care of themselves to avoid those situations that they fear.*” (Nurse, Chronic diseases service, Health center #6).

A second approach was through emotional support, which includes listening to their patients, talking with them about their feelings, providing some advice, encouraging them to do not break down, and helping them to cope with the stigma, especially for patients with tuberculosis or HIV/AIDS.

*“We have to talk to the patient, talk to them, at least touch their shoulder, encourage them, tell them that they are not the first person who is going through this, that there are so many people going through the same thing and they are living their lives normally.”* (Midwife, HIV/AIDS service, Health center #3).
*“As professionals, we are midwives, but in that moment, we are psychologists, too (…) when we have the opportunity to have them in front of us, after they have broken all the barriers to come to the health center, we have to take that opportunity and try to adapt according to the situation, to the reality we face.”* (Midwife, antenatal care service, Health center #1)

This emotional support occurs specifically when PHCPs perceived their patients to be sad or worried, at the beginning of the treatment (mainly for patients with tuberculosis and HIV/AIDS) or when patients themselves decide to talk about their problems or feelings.

In terms of the actual service provision, clinical practice guidelines prescribe at least one mental health consultation for all these patients during their treatment; however, some patients may never effectively see a psychologist - indeed, some patients reported not knowing how to schedule an appointment with a psychologist. PHCPs also informed that they refer their patients to the psychologist only when they perceived them as emotionally affected, usually when they cry during the consultations or exhibit signs of domestic violence, drug abuse, sadness, suicide, etc. In some cases, PHCPs from the chronic diseases service report having also recommended their patients to attend the complementary medicine service to receive alternative therapies that may help them to relax and interact with others.

PHCPs mentioned some limitations to take care of their patients’ mental health. First, they recognized not having the necessary skills or knowledge to deal with their patients’ mental health, so they avoid asking too much about their emotional issues unless patients bring them up. In these cases, PHCPs mentioned trying to provide emotional support and recommending seeking mental health care. Second, PHCPs stated no following up on their patients due to time constraint, so most of the time they are unaware of whether their patients sought mental health care. Third, some PHCPs try to provide a comfortable environment in which their patients can feel at ease and trust them, but this demands time from health providers but also from patients, who may at times be in a hurry to get back to work or other demands on their time, making it difficult for PHCPs to provide support or guidance. Finally, nurse technicians stated having less availability to talk to their patients compared to nurses, due to additional administrative tasks required by their role.

### The provision of psychology services care at the primary care level

***Perspective of psychologists about the care provided in their health services***. According to the interviewed psychologists, mental health services provide a series of activities for the population they serve: screenings for domestic violence, depression, or alcohol and drug abuse; group activities to adolescents, elderly people, or pregnant women; and also informative talks in schools or in the waiting rooms of their health center. Despite this diversity of tasks, they acknowledge their main activity is individual consultations. Interviewees stated most of their patients to be women and young people, who seek help mainly for domestic violence, depression and anxiety. They estimate that 2 in 3 patients schedule an appointment by their own accord, and that others arrive recommended by other health worker. This recommendation, however, is not included in the patient’s record, but merely a face to face suggestion or a piece of paper that reminds patients to schedule an appointment.

Regarding the content of consultations, psychologists commented that a first session usually consists in identifying the problem and providing guidelines to solve it. This rapid response is offered because, despite most patients being advised to return for a follow-up session, most of them do not attend, which leads the psychologist to offer as much help as possible in a one-off consultation. Furthermore, three psychologists stated that most consultations do not include standardized tests due to the limited time allocated (20 minutes that sometimes they try to extend to 45), thus relying their diagnosis exclusively on conversation and their previous experience with similar cases. It was also found that each psychologist had established their own way to provide treatment. For instance, one of them mentioned that he sought to offer Gestalt psychotherapy due to his prior training, yet the other interviewees declared not following any specific theoretical framework or using techniques from different ones.

*“From the theoretical approach, you are almost always asked to conduct an interview, observation, to make an evaluation through instruments and finally arrive at a diagnosis and start the treatment. The practice (however) demands you (to act) in a totally inverse way. The practice is “today I want you to solve my problems, today I come with a certain level of despair, anguish, I need to get out”. So, I think it depends on your skill for the interview, which is what I do, (...) trying to capture the core problem and trying to help, being aware that the patient is not going to return, but for a second or third time and no more.”* (Psychologist, Health center #4).

Depression was reported as the most common problem found in their consultations. However, despite the existence of a MoH protocol to treat depression in primary care, psychologists reported not using it because: 1) they consider it unclear, i.e. one psychologist informed that the protocol signals to refer patients to the psychiatrist and “provide support through a psychological consultation” (Psychologist, Health center #4), without specifying how to provide that support, 2) it is not suited to their context, resources and training, i.e. the protocol indicates administering questionnaires for which they have not received training, 3) they cannot access said protocol, or 4) they were not aware of its existence.

To assess depressive symptoms, psychologists mentioned using different psychometric tools (i.e. Self-Reporting Questionnaire, Goldberg Depression Test), observation, interviews or a combination of these. Some mentioned that they sought indicators (i.e. sadness, inability to manage the problem) to assess the severity of depression. Nonetheless, one psychologist was unsure of how to assess the severity of cases and questioned if the way he assessed them was correct: he considered “between mild and moderate” when symptoms were “more serious” (Psychologist, Health center #2), based on physical symptoms (e.g. problems with sleep, changes in appetite or unwillingness to work). The interviewee reported referring those patients to the psychiatrist.

Reported strategies for treatment of mild depression included listening to the patient, assessing the causes of the problem, providing advice, or referring to a general practitioner or psychiatrist to get pharmacological treatment. Additionally, some reported conducting therapies—e.g. Gestalt therapy, laughter therapy, “companionship therapy” (Psychologist, Health center #1)—, or recommending certain foods to make patients feel better (fruits, condensed milk), or to relax (chamomile). For moderate and severe depression, psychologists informed referring their patients exclusively to the psychiatrist, recommending to attend further sessions, and talk to the family to provide support to the patient. Finally, when suicide risk is identified, psychologists mentioned that they refer to the emergency room, do not leave the patient alone, and try to contact the family. Psychologists also mentioned a lack of collaboration between psychologists and psychiatrists. Since psychiatrist do not usually work in primary care but in hospitals, they barely see each other, due to which psychologists are often unaware of what treatment is being offered by the psychiatrist, or if patients have even arranged an appointment.

Interviewees identified two important facilitators to their provision of mental health care. First, primary care patients are supposed to attend all primary care health services (i.e. nutrition, odontology, psychology), which favours to schedule an appointment with psychologists. And second, the low price or even free-consultation offered to patients who have a public health insurance, as the Seguro Integral de Salud, SIS (Integral Health Insurance). In Peru, this health insurance is provided by the MoH to underserved populations, with a limited number of free appointments for each health service. Regarding barriers to mental health care, psychologists identified some related to the health system. First, a lack of qualified personnel, since most health centers have only one psychologist among their staff, and there is no continued training programs to update their knowledge and skills. Second, some interviewees considered the scheduling mechanisms as a barrier, because according to the care level, some health centers are only open in the mornings, and yet most patients have more available time in the afternoons. Third, the timeframe between appointments in the health center of EsSalud, where despite the supposed benefit of being able to program appointments in advance, the 20 or 30 day anticipation will lead patients to forget their appointment or simply find it difficult to get time off work for a second appointment. Finally, despite some groups of patients are required to have an appointment with a psychologist as part of their integral treatment plan, some psychologists declared not being aware of this.

A second group of barriers relates to patients beliefs regarding mental health care. For example, psychologists believed some patients do not feel the need to visit mental health services for a second time once they have already had a consultation. Interviewees think it relates to patients finding recommendations from a one-off consultation helpful enough, not requiring more help. On the other hand, psychologists mentioned that some patients have never attended a mental health service due to a general misconception of mental health care being only for “crazy” people (people with mental disorders, which are usually stigmatized), believes that their problems are an unchangeable part of who they are (“ *[Patients often say] I am like this and nothing will make me change*” (Psychologist, Health center #4)), or because it is not a priority for them.

Finally, psychologists were in favour of the possibility that PHCPs administer a mental health screening to their patients, since it would ease the early detection of mental disorders, raise awareness of mental health care importance among patients, provide more human resources trained to detect mental disorders, and introduce a procedure that is part of primary care tasks. However, two psychologists were concerned about shifting a task that is part of their professional background, since it could result in less job opportunities for psychologists in primary care:

*If you give this (training in a mental health screening) to the nurses, they would be well trained but new psychologists will not be hired. There would be the risk that in time, the institution says “no more psychologists, only nurses will stay”* (Psychologist, Health center #6).

Likewise, interviewees agreed with the idea of PHCPs referring detected patients to psychology services because this will allow patients to receive opportune treatment and help psychologists to increase their productivity (which could be positive since their performance is usually assessed based on their productivity). Nonetheless, they were worried about not being able to tend the entire demand of patients that would be referred, due to the scarcity of human resources and few time allocated per consultation. One psychologist mentioned, however, that this could show the lack of psychologists in primary care, resulting in hiring more psychologists.

***Perspective of PHCPs about care provided in the psychology services***. PHCPs from the different services agreed that the care offered by the psychologists was good for patients and effective in promoting wellbeing. Moreover, they described how their services collaborate with the psychology service to offer workshops or presentations in order to promote their patients’ mental health or to motivate their adherence to treat their physical conditions. These activities, however, are not developed continuously due to the personnel often not getting along or due to other responsibilities. Nonetheless, whenever they have had the opportunity to work together, both PHCPs and psychologists have thought of it as a positive experience and valuable to their patients.

On the other side, PHCPs considered psychologists to have very limited time per consultation, which ultimately reduced the quality of care provided. Besides, since psychology services are due to tend to all patients from the health care centers, they are unable to cover the entire demand. Finally, some PHCPs mentioned that psychologists do not ask their patients to return for following sessions, which takes PHCPs to continuously refer them to seek additional sessions with the psychologist if they deem their patients to need it.

***Perspective of patients about the care provided in the psychology services***. Most interviewed patients (31/37) declared having attended to a psychologist at least once in their lifetime. Twelve stated their first ever contact with a psychologist to have been as part of their physical condition’s treatment, usually at the beginning. Six mentioned this first contact was related to their children’s school psychologist and four when they were young, in their school, university or work. Despite most patients interviewed deemed important to seek mental health care, most of those who have ever attended, stated doing so only because they were required to. Only a few declared seeking mental health care of their own accord.

Some patients mentioned certain barriers to seek mental health care: time to attend the health center, since they usually spend an entire morning waiting to receive care; uncertainty of whether the consultation would be expensive or free; and lack of knowledge of the procedures to arrange an appointment. Additionally, despite most patients interviewed did not consider stigma as a barrier for them, they do recognized that some people think that psychologists are for “crazy” people: *“We need psychological help, even though many times it is demonized and people say ‘No, I am not crazy to go to a psychologist’”* (Patient, Chronic diseases service, Health center #6).

The general opinion among patients interviewed is that the psychologists are helpful in offering emotional support and they acknowledge psychologists as professionals who can provide advice and guidance. Most of those who had indeed sought mental health care at least once in their lifetime, stated that they were satisfied with the care provided and considered the psychologist’s recommendations to be useful. Taking a closer look, we found some differences among health care services. For instance, in antenatal care services, patients informed that their consultations with the psychologists from their health centers consisted of questions regarding how they were feeling, recommendations for a good pregnancy and relationship advice. In the tuberculosis services, patients mentioned that the main focus of mental health care was to assess the impact of the physical health condition on their emotional wellbeing, and on reinforcing their adherence to the anti-tuberculosis treatment. In the HIV/AIDS services, patients reported that the psychologists provided emotional support, through listening to them or trying to understand how they were feeling when they were diagnosed or when they were feeling sad; helped them to accept their physical health condition, as typically a first reaction is of denial; and reinforced the importance of following their antiretroviral treatment. Finally, in the chronic diseases service, patients stated that the psychologist offered advice to better control their physical condition, such as avoiding stressful events and improving relaxation; recommendations to improve their lifestyle, like doing exercise and eating healthy; and guidelines to solve problems including low self-esteem or depression.

Six participants, however, had negative opinions regarding the care received. They explained that they did not like the recommendations provided, the psychologist’s rude attitude, they were uncomfortable to share their personal experiences, or they felt that the psychologist did not understand them.

*“He did not understand me and everything was very quickly, it seemed that the man (psychologist) was hurry and did not try to make me talk (...) I did not come back, just went (to that appointment) because (the PHCP) told me that I had to go to the psychologist.* (Patient, Tuberculosis service, Health center #6)

Half of interviewed patients stated that they were recommended to seek mental health care by their PHCPs. Moreover, it is noteworthy that many patients reported having established a close bond with their PHCPs, thus also trusting their opinion and usually follow their recommendations. Therefore, they expressed a good disposition to seek mental health care if their PHCPs advised so.

“ *If the doctor tells me that I need to go to a psychologist, then I would go because there should be a reason and I think that doctors are right*” (Patient, HIV/AIDS service, Health center #5)
*“The nurse from the service advised me, ‘you have to go to the psychologist’ she said, because I told her about my problem with my husband, ‘go to the psychologist and talk with him’. That is why I went to the psychologist, since she told me”* (Patient, Tuberculosis service, Health center #6).

## Discussion

We proposed to understand the current offer of mental health care at the primary care level, collecting the views of psychologists, PHCPs, and patients. We confirmed a high perceived need of mental health care in primary care patients’, as found in other low- and middle-income countries ^24^. However, despite the awareness of the importance to attend primary care patients’ mental health and the activities conducted by PHCPs and psychologists to address it, there are significant limitations in the care provided. Below, we discuss the main findings, organized by groups of participants.

Among PHCPs, we can summarize our findings into five key messages. First, PHCPs from all health services perceived an emotional impact of the physical health condition on their patients, as found in other studies ^25– 28^, thus requiring mental health care. However, it is important to ask whether this perception is then translated into a prioritization of mental health or, the opposite, to a normalization of the emotional impact, therefore potentially leading PHCPs to fail in adequately address it. Therefore, it would be important for PHCPs to receive training regarding their patients’ mental health, including the emotional impact of the physical condition, detection of mental disorders in their patients and referral of positive cases to mental health care. Second, most interviewees noted that even when they don’t initiate the conversation, some patients spontaneously share their feelings and problems with them, as found in the literature ^29^, thus PHCPs try to provide emotional support (listening, talking about their feelings, provide advice, etc.), even when they do not have the appropriate time and training to do so.

Third, despite PHCPs declaring usually recommend their patients to attend to the psychologist, they also noted to only insist with those patients who appear to need it the most and are ultimately unaware of whether their patients sought this care. Fourth, PHCPs are not usually trained in mental health, so their decision to provide advice or refer patients to mental health services might be largely based on their own subjective opinion and common sense rather than on a standardized criteria. This is an important point of concern, as evidence suggests that PHCPs do not always correctly identify common mental disorders in their patients ^12^. Evidence does suggest however that PHCPs with adequate training and supervision are fully capable to detect, diagnose, treat, and monitor people with mental disorders ^30^.

Finally, the health system’s limitations, particularly the scarcity of psychologists operating in services overwhelmed by patients, are translated in short consultations and with less frequency than required to guarantee adequate mental health support. This shortage of psychologists, a problem found in many middle-income countries ^31^, could be addressed through task-shifting, in order to provide a more efficient use of the existing human resources ^32^. This is what the MoH aims for with the Peruvian Mental Health Reform, through the implementation of the mhGAP ^33, 34^ and the common mental disorders’ screening conducted by PHCPs ^9, 10^. The current provision of emotional support that PHCPs referred offering could be an important facilitator for this task if appropriate training is provided; but might be also limited by the resistance among health professionals to change their practices into a new model of care ^35^, as psychologists’ concerns with shifting the screening task to PHCPs.

In the case of psychologists we have four significant findings. First, they prioritize facility-based instead of community based activities, even though they are supposed to provide both ^36^. This prioritization is made through one-to-one consultations, due to the high number of patients demanding treatment and recovery care. This situation is also reported in other countries ^37^, leading psychologists to relegate prevention and promotion activities, which limits the reach and potential of primary care actions at the community ^38^. Currently, the ongoing Peruvian Mental Health Reform has changed towards a community model of care, which might address this limitation by implementing Community Mental Health Centers (CMHC) across the country. These CMHC provide specialized mental health care at the primary care level, combining facility-based activities and community-based ones, as home visits and community programs.

Second, as in several Latin American countries, psychologists do not follow a standardized model of care ^39^. Despite the existence of a depression treatment protocol, it is not used either because psychologists do not consider it appropriate —probably because it emphasizes pharmacological treatments, which they cannot prescribe, rather than interventions that can be provided by psychologists, as counselling or psychotherapy, which are mentioned but not deeply explained ^40^—, they do not have access to it, or they do not know of its existence. Thus, each psychologist provides different treatments and states to usually refer their patients to psychiatry, which is indicated in the guideline ^40^. Additionally, despite the existence of effective treatments for depression in low- and middle-income countries ^41– 43^, it is concerning that all psychologists offer different treatments, which are not necessarily evidence-based and that may be inconsistent across patients, services and health facilities.

This brings the role of the MoH into discussion with regards to seeking to provide the best possible care and prioritizing evidence-based care ^44^; as well as the role of patients, who do not attend to more than one session, making it difficult for psychologists to provide an appropriate treatment. Since there is a protocol that is currently promoted by the national health system, but psychologists do not use it, some actions must be taken to address this gap. Based on the barriers reported by the psychologists, it would be advisable to provide printed guidelines to primary care psychologists, train them on its use, including the tests, treatments and procedures of referral that they must follow, and offer alternatives to adapt the contents to their contexts. Likewise, recommendations for future guidelines may involve the incorporation of evidence-based pharmacological and non-pharmacological treatments, assure the provision of such guidelines, and the corresponding training to the health workers that are supposed to implement them.

Third, another system-level barrier includes the shortage of psychologists in the health services, their restricted availability to work only in the mornings, the long waiting periods between appointments, affecting adequate monitoring and follow-up, and unawareness that patients from other health services are eligible to attend to their service. Furthermore, despite being crucial for primary care psychologists to receive training specific to their role -focusing on prevention, collaborative work, knowledge of psychopathology, research skills ^45^-, psychologists do not receive continuous training, so update depends on their own willingness. Besides, psychologists also mentioned the limitations from the SIS to cover mental health treatment in primary care, and the inconvenience that some health centers are open only in the mornings. Nowadays, the current Peruvian Mental Health Reform has increased the SIS budget to extend its coverage, which has been translated into more consultations ^46^; and primary health care centers have extended their schedules to also work in the afternoons ^47^. Now, it would be important to not only extend the coverage and health centers’ schedules, but to motivate patients to attend continuously.

Fourth, limited integration and mutual support to conduct collaborative work, as found in other Latin American countries ^39^. Among PHCPs and psychologists, we found that this collaboration is only through referrals or few activities conducted co-jointly, which may limit the possibility to provide a more integrated mental health care across services to those who need it the most and remain underdiagnosed inside primary health care services. Regarding psychologists and psychiatrists, by the time in which the study was conducted, primary care psychologists refer their patients to psychiatrists working at the secondary and tertiary care level, where there were no communication channels to coordinate the care provided, possibly meaning an overlapping of advice and treatment, or even opposite guidelines offered to the same patient. Nowadays, psychiatrists are also found in primary care due to the current implementation of CMHCs. This situation might ease the communication among these professionals, following the recommendation of the WHO, which states that it is important to improve the collaborative work among health providers in order to provide quality care ^48, 49^.

Finally, for the group of patients we have four main findings. First, we found that their most frequent first contact ever with a psychologist was as part of their physical condition’s treatment. Thus, the referral made by PHCP to psychology services might be facilitating the access to mental health care, which would be highly important to detect and treat cases that might need such care. Moreover, an important finding was the patients’ willingness to seek mental health care if recommended by their PHCPs, which optimistically points to patients’ potential receptiveness towards mental health care.

Second, patients help seeking behavior. We found that despite the health system proposing and PHCPs stating they usually recommend their patients to attend to the psychology service, only half of the patients interviewed mentioned having received such recommendation, and psychologists estimated that only 1 in 3 patients attended by recommendation of a PHCP, thus psychologists and patients show that not all patients who are recommended finally access the psychology services. This confirms the low percentage of people in Peru who seek mental health care, as reported in previous local studies ^3, 50^.

Third, this patients’ low attendance to psychology services might be explained by a series of barriers. First, the procedures to arrange an appointment, which demand several hours of the patients’ time. Second, the lack of knowledge regarding the consultations costs. Nowadays, consultations in mental health care are covered by the SIS ^46^, thus, patients must be informed, in order to motivate them to seek care and access to treatment. Third, and as in other countries, we found attitudinal barriers from patients, as considering that they can solve the problem by themselves, see the treatment as ineffective or having negative experiences with the mental health providers ^51^. Therefore, it is advisable to work in those attitudinal barriers, which might be also found by the Peruvian Mental Health Reform, in order to improve the access to treatment in mental health care. Thus, strategies aimed to provide information about mental health care access and to reduce stigma should be encouraged.

Fourth, most patients who attend to a psychologist were satisfied with the care received. They highlighted that psychologists tried to assess the impact of the physical conditions over their mental health, receiving useful guidelines to adhere to their treatments and change their lifestyles. This would signal that psychologists are seeking to provide an integrated health care responsive to other health conditions needs. Nonetheless, as patients usually attend only one consultation with psychologists, they probably only receive advice and recommendations. If the number of sessions increase and the adherence of patients to mental health care improve, psychologists might enhance the guidelines they already provide, which might be translated into a better care of patients’ health conditions.

Finally, we also found that some patients complained about the psychologists’ rude attitude and lack of empathy, which highlights the importance of assessing the quality of care provided. This assessment would be valuable for the implementation of the Peruvian Mental Health Reform in primary care services in order to improve the quality of the care provided and its responsiveness to patients’ needs.

Our findings showed how mental health care is provided in primary care, by both PHCP and psychologists, and show some situations that should be taken into account in order to improve the current provision of mental health care and its integration in primary care through the Peruvian Mental Health Reform. First, it is essential to secure that PHCP and psychologists are able to conduct their corresponding tasks appropriately, receiving an appropriate training, supervision, and having all the necessary materials and resources to do so, which we have found as weaknesses in primary care. Second, there is a need to reduce the diversity of treatments provided by primary care psychologists to those that are evidence based, including non-pharmacological treatments in the national guidelines related to mental health care. As stated before, it is necessary to provide appropriate training and supervision to mental health providers to ensure the correct implementation of the protocols. Third, it would be important to strength the collaboration among PHCP, psychologists and psychiatrists across the public health system. Our results demonstrated that there is little collaboration among these health professionals, which might limit the efforts of the Peruvian Mental Health Reform regarding the integration of mental health care into primary care. The current implementation of CMHCs might help overcome this limitation since they are based on team-based interdisciplinary work. These teams also provide technical support and supervision to primary health care services ^52^. Finally, it is advisable to incorporate strategies that address the mental health treatment gap, including the help seeking behavior and access to mental health care for patients who need it, as well as their adherence to the treatment provided. We found that a major barrier for improving the quality of the mental health care provided was not only the limitations of the health system but also the attitudes of patients, who were not used to seek mental health care and usually attend no more than one or two appointments with psychologists.

Finally, this study’s limitations should be duly noted. Whilst we achieved a sufficient variety of PHCPs, the number of psychologists reached was quite low, not by choice, but significantly reflecting the current shortages in human resources within mental health services. However, we were able to collect and analyze relevant information, which enabled us to understand how mental health care activities and services were provided in their health centers. Secondly, there may be specific biases particularly information and social desirability bias, yet the qualitative data retrieved was diverse in terms of opinions and experiences regarding the mental health care in primary care. This information allows for a better and richer understanding of the health system dynamics at the primary care level in Peru with an emphasis on the provision of mental health care across a number of services where continuous care is needed, together with an adequate assessment of barriers and facilitators to provide quality health care.

The current provision of mental health in primary care in Peru remains a challenge. As we have observed, mental health care is, in a certain way, already provided by both PHCPs and psychologists. Understanding both barriers and facilitators, and identifying opportunities to provide adequate mental health care at the primary care level will offer guidance toward simple, efficient and low-cost solutions to improve the current situation.

## Data availability

The audio and transcripts of the interviews contain identifiable and sensitive information. For that reason and to protect participants’ confidentiality, the interviews have not been made available. To apply for access to the data, please contact the authors at the institutional email address of CRONICAS Center of Excellence in Chronic Diseases ( cronicas@oficinas-upch.pe). Data, however, will only be shared if certain conditions are met. First, those who apply to access the data are obligated to maintain the confidentiality of the data under any circumstances. Second, they will not seek to identify any individual or organization mentioned in or related to the study data; and if they discover the identity of any individual or organization, they must not use this information but immediately contact the authors of this article. Third, the authors of the article must be informed about any publication (paper, theses, dissertations, presentations, among others) in which the data will be used, prior to their dissemination. Finally, if the data is used in any other publication, it should be acknowledged that it is of secondary source, providing the appropriate citation. 

Please note that the audio and transcripts of the interviews are only available in Spanish.

## Supplementary material

Supplementary File 1: Codebooks developed for the analysis of the data.
