# Supplementary material for: Provision of mental health care within primary care in Peru: A qualitative study exploring the perspectives of psychologists, primary health care providers, and patients
Source: Wellcome Open Res. 2018 Apr 30;3:9. Originally published 2018 Feb 12. [Version 2] doi: 10.12688/wellcomeopenres.13746.2 (PMC5861510; doi:10.12688/wellcomeopenres.13746.2)
Supplement: Supplementary file 1 [file wellcomeopenres-3-15868-s0000.tgz › 5d062300-f9b5-453a-8bab-dc62b7d5298f.docx]

## Supplementary File 1

## Codebooks developed for the analysis of the data

Codebooks were developed in order to guide the analysis of the data, conducted by three different members of the research team. These codebooks were based on the consensus of the most relevant topics of one selected interview, and all the following interviews were coded using these codebooks. The research team developed three codebooks: One for psychologists, other for PHCPs, and other for patients. All these codebooks had a definition of each code which ease the familiarization with the contents and the analysis of the data.

**A - Codebook for psychologists’ interviews**

**A1. Characterization of the mental health service:** Description of the patients who attend to the mental health service and their adherence; the care provided by the psychologists; the care provided to treat depression; and the limitations of their health centers to provide a better mental health care.

**A2. Access and referral of patients to the mental health service:** This includes how patients attend to the mental health service (i.e. by their own, referred by other health provider); and facilitators and barriers to receive mental health care.

**A3.** **Co-joint work with other health services:** Includes the way in which psychologists work with other health services.

**A4. Importance of mental health:** Opinions about the importance that other health workers and the health center gives to the mental health care.

**B – Codebook for PHCP’ interviews**

**B1. Patients’ experiences:** Information provided by PHCPs about their patients. This include how the physical health condition impact their patients’ lives, the idea that patients have about mental health, depression and psychologists, and the experiences that patients have had with the psychologist of their health center.

**B2. Perception of mental health:** Perceptions of PHCPs about their patients’ mental health and the impact of their physical health condition on their mental health; the mental health care needs of their patients; and how they detect cases that may require specialized mental health care.

**B3. Actions in front of patients’ need of mental health care:** What PHCPs usually do to support their patients to manage their physical health condition and their emotional wellbeing; and cases in which the PHCP had identified a need of emotional support, including the actions taken in those cases.

**B4. Care provided in the health service:** It includes the way PHCPs treat their patients; their perceptions about the conditions of the health service (infrastructure, functioning, care provided); the capacity of PHCPs to deal with their patients’ mental health; and the training received in different topics, including who, when and why it was provided and the utility of such trainings.

**B5. Co-joint work with other health services:** Includes the way in which the different health services work together; the work with the psychologist; and their opinion about the psychologist of their health center.

**C – Codebook for patients’ interviews**

**C1. Impact of the health condition:** Impact that the health condition has in the different domains of patients’ life, with a higher emphasis in the negative consequences. It includes the positive impact, the negative impact (emotional wellbeing, relationships, work or studies, and others), and no impact.

**C2. Idea of mental health:** Notions that patients have about depression and the psychologist as a mental health professional.

**C3. Resources to cope with the emotional distress:** Resources include all the actions that patients take to feel better, considering those with and without interaction with others. These actions are grouped in personal resources, seeking mental health care from a psychologist, receiving support from their PHCP, support from friends and family, or support from other people/groups (religion). All these resources include the motives to seek or receive care and the results/opinion of receiving such care. The barriers encountered to cope with the emotional distress are also included.

**C4. Experience in the health service:** Appreciations of patients regarding the health service (infrastructure, functioning), and description and opinions of the care received from the PHCPs.

**C5. Experiences of other people:** Experiences told by patients but not directly related to them. I.e. experiences they heard from others.

**C6. Past (previous to the health condition):** Experiences that occurred to patients before having the physical health condition.
