# Supplementary material for: Provision of mental health care within primary care in Peru: A qualitative study exploring the perspectives of psychologists, primary health care providers, and patients
Source: Wellcome Open Res. 2018 Apr 30;3:9. Originally published 2018 Feb 12. [Version 2] doi: 10.12688/wellcomeopenres.13746.2 (PMC5861510; doi:10.12688/wellcomeopenres.13746.2)
Supplement: Peer review history

# Peer review: Referee response for version 2

**DOI:** 10.21956/wellcomeopenres.15868.r33022

**Published:** 2018-05-04

**Authors:** Rubén Alvarado

**Affiliations:** 1 Institute of Health Sciences, Universidad de O'Higgins, Rancagua, Chile; 2 School of Public Health, Faculty of Medicine, Universidad de Chile, Santiago, Chile

Two of the three critical observations were answered, and the third had only a partial response.

 The changes are sufficient to accept the publication.

 This second version is approved.

I have read this submission. I believe that I have an appropriate level of expertise to confirm that it is of an acceptable scientific standard.

# Peer review: Referee response for version 1

**DOI:** 10.21956/wellcomeopenres.14937.r30766

**Published:** 2018-03-19

**Authors:** Rubén Alvarado

**Affiliations:** 1 Institute of Health Sciences, Universidad de O'Higgins, Rancagua, Chile; 2 School of Public Health, Faculty of Medicine, Universidad de Chile, Santiago, Chile

It is a good job and makes a valuable contribution to understanding the process of mental health reform in your country (Peru), which can be projected to the situation of mental health care in primary care centers of other countries with similar characteristics.

 I recommend its indexing, asking the authors to improve three topics:

 - to argue why the results in the centers that were included could be similar in primary care centers throughout Peru;

 - to describe in more depth the method of analyzing the discourses of the interviews (emerging categories, etc.); and,

 - deepen the explanation of the professionals for not using clinical guidelines or protocols; because this is important for possible strategies that use screening tools or guidelines such as WHO's MHGap.

I have read this submission. I believe that I have an appropriate level of expertise to confirm that it is of an acceptable scientific standard, however I have significant reservations, as outlined above.

# Peer review: Referee response for version 1

**DOI:** 10.21956/wellcomeopenres.14937.r30767

**Published:** 2018-03-02

**Authors:** Saroj Prasad Ojha

**Affiliations:** 1 Department of Psychiatry & Mental Health, Institute of Medicine (IoM), Tribhuvan University Teaching Hospital (TUTH), Kathmandu, Nepal

I found the article comprehensive in terms of literature review, methodology was sound, clear and precise, and being a more descriptive study there was more focus on describing qualitative results. The summary was to the point in relevance to the title of the paper.

 Some additional inputs are:              

 Introduction: If the author could find and add literature about acceptance and attitude towards mental illness among people visiting PHCP.

 Methods: Justification of use of centers has scope of elaboration.Why these centers?

 Ethical statement: the date of IRB approval is missing?

 Results: For a qualitative study the result is broad and comprehensive and fulfills the objectives in the study methodology.

 Discussions: One concern is why were psychiatrists not a part of the study should be mentioned in discussion. Is it to do with the health structure system or why were they excluded all together from the study? Otherwise, the discussion including the limitation and conclusion are in context to the study design, objective and title.

 The discussion could also make some elaboration on the policy implication of the research finding on the existing health scenario.

 In this context, it is a good study well written, and after review of the article, I believe that this article is worthy.

I have read this submission. I believe that I have an appropriate level of expertise to confirm that it is of an acceptable scientific standard.

# Peer review: Referee response for version 1

**DOI:** 10.21956/wellcomeopenres.14937.r30836

**Published:** 2018-02-19

**Authors:** Andrea Bruni

**Affiliations:** 1 Pan American Health Organization (PAHO), Washington, DC, USA

A research on the integration of mental health into PHC in Peru is very much welcome.  I also find interesting the identification of antenatal care, TB, HIV/AIDS and chronic diseases clinics for conducting the research.

 However, the paper makes no reference at all to the WHO Mental Health Gap Action Programme (mhGAP),  which is the global programme on integration of mental health into PHC. It seems surprising that a paper  on the integration of mental health into PHC doesn’t consider this very important programme. Importantly, mhGAP programme is also being implemented in Peru. 

 The paper makes a vague reference to the Peruvian mental health reform that has been ongoing over the past few years. However, this is very superficial to the extent that doesn´t take into account of community based mental health centers *(Centros de Salud Mental Comunitaria, CSMC*) which belong to PHC.

 The huge treatment gap existing in the country, is entirely explained  ‘ *by the insufficiency and inequitable distribution of resources, the small budget allocated to mental health, and the poor integration of mental health care into primary care’. * This seems simplistic to me.

 I also suggest to use consistent terminology throughout the paper; for instance services users are sometimes referred to as *patients* and some others as *users of primary health care centers*.

 It doesn´t seem very clear why in the paper psychologists deployed at PHC level do not belong to the category of PHCPs.

 It would have been interesting to explore the impact of mental health interventions on adherence to treatment in persons with TB and HIV/AIDS.

 The population of ‘patients’ was the largest (37 patients vs 4 psychologists and 22 PHCPs), despite this, the section dedicated to this population in the discussion seems to be extremely scanty.

I have read this submission. I believe that I have an appropriate level of expertise to confirm that it is of an acceptable scientific standard, however I have significant reservations, as outlined above.
